# Pedigree analysis of cerebral venous sinus thrombosis associated with compound heterozygous hereditary protein S deficiency

**DOI:** 10.1007/s00277-026-07153-3

**Published:** 2026-07-06

**Authors:** Xuanyu Chen, Beilei Hu, Buyu Ke, Chenyu Dai, Xinying Li, Chuyi Wu, Ming Zou

**Affiliations:** 1 Department of Neurology, The Second Affiliated Hospital and Yuying Children’s Hospital of Wenzhou Medical University, Wenzhou, 325003 China; 2 Wenzhou Key Laboratory of Neurogenetics, Wenzhou, China

**Keywords:** Hereditary protein S deficiency, Cerebral venous sinus thrombosis, *PROS1*, Compound heterozygous variants

## Abstract

This study investigates the relationship between hereditary protein S deficiency (PSD) and cerebral venous sinus thrombosis (CVST) through phenotypic and genetic analyses in a proband and family members with compound heterozygous *PROS1* mutations. A retrospective analysis was conducted on the clinical data of one patient diagnosed with CVST treated in The Second Affiliated Hospital and Yuying Children’s Hospital of Wenzhou Medical University in July 2025. Peripheral venous blood samples were collected from the proband and 14 individuals from three generations, and relevant coagulation parameters and thrombin generation and inhibition assays were performed. Plasma protein S activity (PS: A), total protein S antigen (TPS: Ag), and free protein S antigen (FPS: Ag) were quantified. All exons and flanking regions of PROS1 were amplified by polymerase chain reaction (PCR) and analyzed by Sanger sequencing. Bioinformatics tools were used to evaluate mutation conservation, predict pathogenicity, and analyze structural effects on the protein. Thrombin generation and inhibition assays indicated defective anticoagulant function in the proband. The proband was diagnosed with CVST and type Ⅰ PSD (PS: A 39%, TPS: Ag 53 mg/L, FPS: Ag 42 mg/L). Genetic analysis identified a heterozygous missense variant c.1544G > A (p.Arg515His) and a heterozygous synonymous mutation c.2001 A > G (p.Pro667Pro) in the PROS1 gene. Arg515 was highly conserved across nine homologous species. The p.Arg515His mutation was predicted to be pathogenic and associated with reduced protein stability and abnormal folding. Compound heterozygous variants c.1544G > A and c.2001 A > G in the PROS1 gene may be associated with reduced protein S levels in this pedigree. The occurrence of CVST in the proband with PSD patients may also be related to these compound heterozygous variants.

## Introduction

Cerebral venous sinus thrombosis (CVST) is a rare subtype of ischemic stroke caused by thrombus formation in the dural venous sinuses, intracerebral veins, or both, resulting from multiple interacting prothrombotic risk factors. It primarily affects young women, with an incidence ranging from 1.3 to 2.6 per 100,000 individuals [1]. The most common hereditary risk factors include quantitative or functional deficiencies of protein C (PC), protein S (PS), and antithrombin (AT), as well as inherited prothrombotic genetic variants such as factor V Leiden [2].

**Fig. 1:**
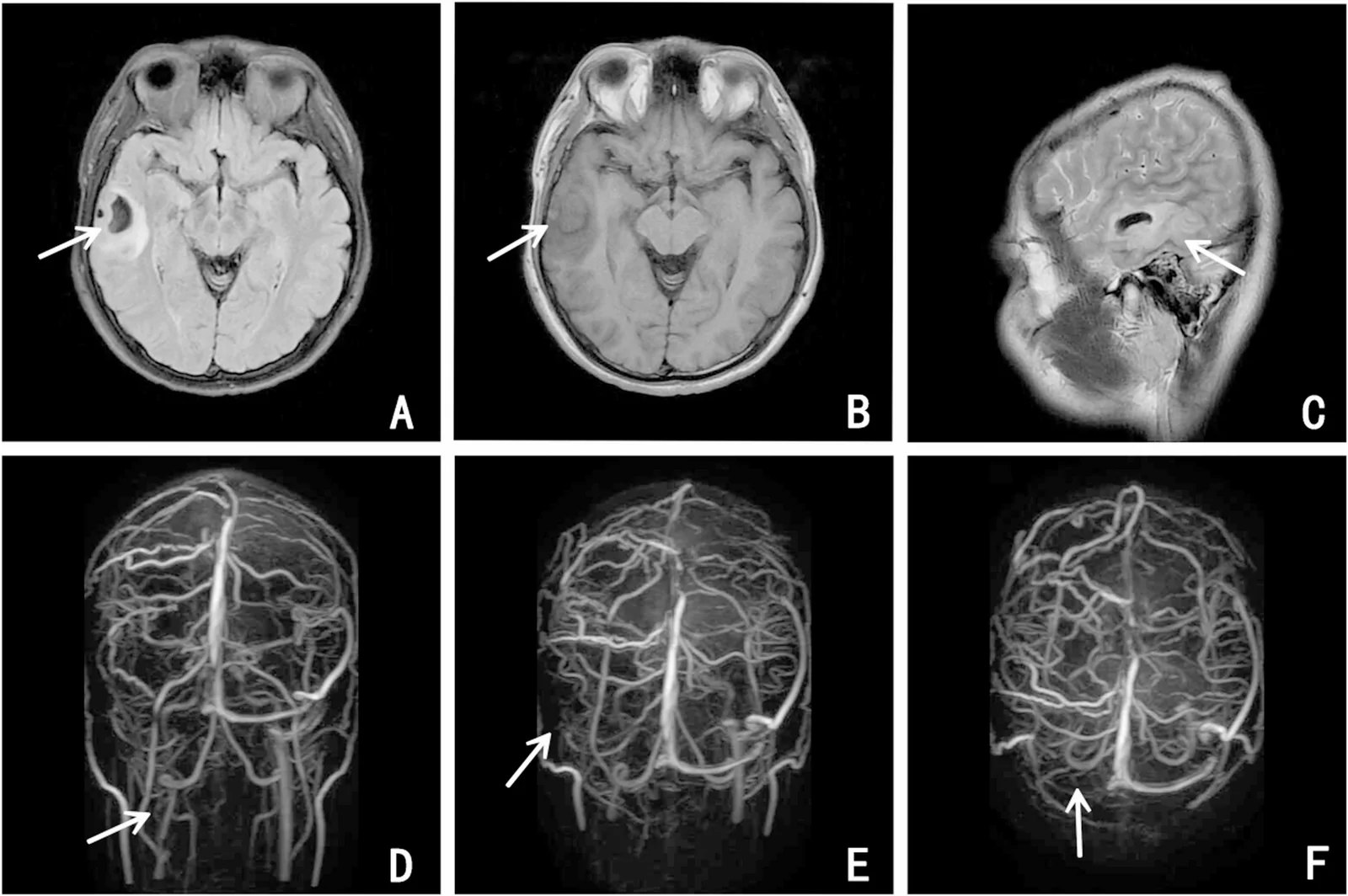
Head MRV demonstrating CVST in a proband with hereditary PSD. Note: **A**: Axial T1-weighted image showing the right temporal lobe infarction (arrow). **B**: Axial T2-weighted FLAIR image showing right temporal lobe infarction (arrow). **C**: Axial diffusion-weighted image showing right frontal lobe infarction (arrow). **D**: Maximum intensity projection (MIP) angiography showing a partial filling defect in the right internal jugular vein (arrow). **E**: MIP angiography occlusion of the right sigmoid sinus (arrow). **F**: MIP angiography showing a filling defect in the right transverse sinus (arrow).

PS is a vitamin K-dependent glycoprotein synthesized in hepatocytes, vascular endothelial cells, and platelet alpha granules. Approximately 40% circulates in free form, functioning as a cofactor for activated protein C (APC) and tissue factor pathway inhibitor (TFPI), suppressing thrombin generation [3]. Inherited protein S deficiency (PSD) is an autosomal dominant thrombophilic disorder caused by pathogenic variants in the *PROS1* gene. Population and family-based studies have demonstrated that inherited PSD is associated with a significantly increased risk of venous thromboembolism (VTE) at a young age [4]. However, intracranial and splanchnic venous thrombosis remain rare manifestations, while systemic VTE is more common. This study reports a case of CVST due to hereditary PSD and investigates genotype-phenotype correlation using family and bioinformatics analyses (Fig. 1).

## Study subjects and methods

### Clinical data

**Fig. 2:**
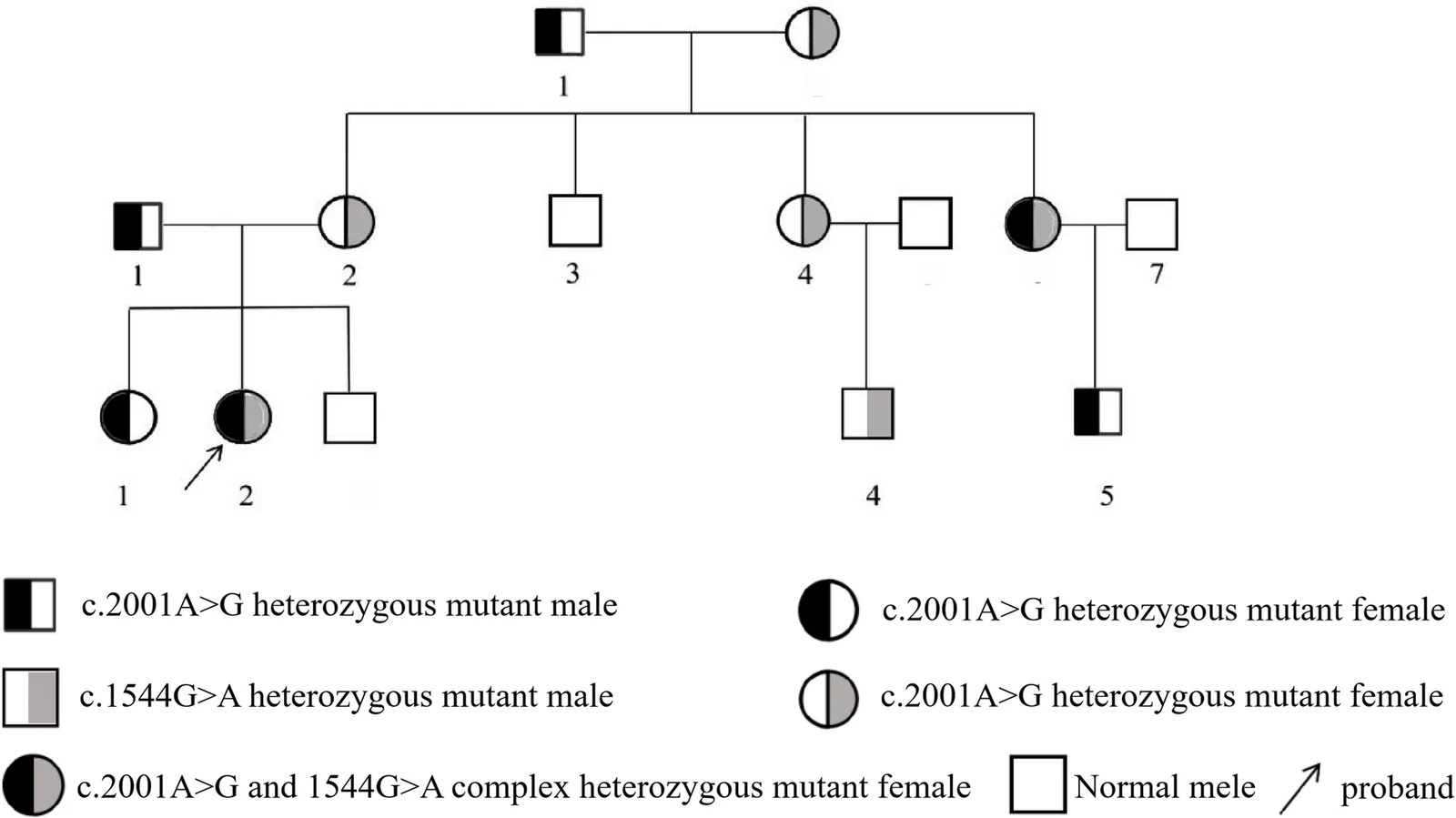
Family pedigree of hereditary PSD

The proband (Fig. 2, III2), a 31-year-old woman, presented to the Emergency Department of The Second Affiliated Hospital and Yuying Children’s Hospital of Wenzhou Medical University in July 2025 with 24-hour persistent cephalalgia and abdominal discomfort. Initial head CT showed a hyperdense lesion in the right temporal lobe, suspicious for hemorrhagic neoplasm. Subsequent MRI indicated venous infarction with hemorrhage, and the patient was admitted. Physical examination revealed stable vital signs and no focal neurological deficits. The patient had a 4-year history of polycystic ovary syndrome and long-term oral contraceptive use, with a VTE risk score of 1. After admission, magnetic resonance venography (MRV) of the head demonstrated non-visualization of parts of the right sigmoid sinus, transverse sinus, torcular herophili, and internal jugular vein, consistent with thrombosis (Fig. 1). Bilateral lower extremity arterial and venous ultrasound findings were unremarkable. Laboratory evaluation showed mildly elevated D-dimer 0.67 mg/L (reference range 0-0.50 mg/L), with normal coagulation parameters including activated partial thromboplastin time (APTT), prothrombin time (PT), thrombin time (TT), and fibrinogen (FIB). Thrombophilia screening demonstrated decreased protein S activity (PS: A 39%; reference range 80%-120%), with normal protein C activity (PC: A), antithrombin activity (AT: A), and plasminogen activity (PLG: A). The patient was treated with low molecular weight heparin (0.2 mL subcutaneously twice daily), mannitol for intracranial pressure reduction, levetiracetam for seizure prophylaxis, and other supportive measures. After 16 days, her condition stabilized, and anticoagulation was switched to oral rivaroxaban (10 mg twice daily). After six months, the patient went back to the outpatient clinic for a follow-up. Once oral anticoagulation has been discontinued for a week, the PS: A, total TPS: Ag, and FPS: Ag were re-measured. The results were consistent with those obtained in the initial laboratory tests. There was no personal history of hypertension or diabetes, and liver and renal function were normal. Family history was negative for thrombotic or hemorrhagic disorders (pedigree shown in Fig. 2).

## Materials and methods

This study adopted the reference range of coagulation indicators, which is routinely used in the daily work of the laboratory center of our hospital. 100 healthy subjects with normal liver and renal function and no history of bleeding or thrombosis were enrolled as controls to verify the reference ranges and exclude genetic polymorphisms. (mean age 32 years; 55 males, 45 females). All coagulation data of controls fell within normal ranges, and no c.1544G > A (p.Arg515His) variant was detected via genetic sequencing. Ethical approval was granted by the Second Affiliated Hospital and Yuying Children’s Hospital of Wenzhou Medical University’s Institutional Ethics Committee (Approval No. 2024194), with written informed consent obtained from all participants.

### Specimen collection and processing

Peripheral venous blood (2.7 mL) was collected into 109 mmol/L sodium citrate tubes (1:9 blood-to-anticoagulant ratio). Samples were gently inverted and centrifuged at 3000 rpm for 15 min at 4 °C. Platelet-poor plasma was separated for coagulation analysis, and cellular fractions were used for genomic DNA extraction.

### Coagulation assays

Coagulation activities of PLG: A, AT: A, PC: A, and PS: A were measured using a STA-R-Max automated coagulation analyzer (STAGO, France). TPS: Ag and FPS: Ag levels were quantified using enzyme-linked immunosorbent assay (ELISA) according to established protocols [5]. All analyses were performed in accordance with Clinical and Laboratory Standards Institute recommendations.

### Thrombin generation and inhibition assay

Calibrated automated thrombogram (CAT) analysis was performed to evaluate thrombin generation dynamics according to previously published protocols [6]. Thrombin generation was assessed in pooled normal plasma and participant plasma, with and without soluble thrombomodulin (sTM), to evaluate PC/PS axis-dependent anticoagulant activity. All assays were carried out using the FluoCa reagent kit (STAGO, France) and 20 µL of PPP reagent per reaction, according to the manufacturer’s instructions. Thrombin generation was initiated with 5 pmol/L tissue factor. Peak thrombin concentration (nmol/L) and endogenous thrombin potential (ETP, nmol/L·min) were calculated from thrombin generation curves.

### Genomic DNA extraction and sanger sequencing

Genomic DNA was extracted from the peripheral whole blood of the proband using a commercial DNA extraction kit (Tiangen Biotech, Beijing, China). Primers targeting the *PROS1* gene were designed using Primer 5.0 software, covering all 15 exons, flanking regions, and 3’ and 5’ untranslated regions. Primers were synthesized by Shanghai Shenggong Biotechnology Co., Ltd., based on published sequences [7]. PCR amplification was performed according to the manufacturer’s instructions under the following cycling conditions: initial denaturation at 95 °C for 5 min, followed by 35 cycles of denaturation at 95 °C for 30 s, annealing at 58 °C for 30 s, extension at 72 °C for 30 s, and a final extension at 72 °C for 10 min. PCR products were purified by the manufacturer and subjected to Sanger sequencing. Sequencing data were analyzed using Chromas software and aligned with the *PROS1* reference sequence (GenBank accession No. AY192161.1) to identify sequence variants, which were confirmed by bidirectional sequencing. After identification of variants in the proband, targeted PCR amplification and sequencing were performed in family members to verify corresponding sites.

### Bioinformatics analysis

ClustalX 2.1 was used for multiple sequence alignment of the amino acid region surrounding the variant site in human (*Homo sapiens*) *PROS1* and eight homologous species to evaluate evolutionary the conservation of the affected residue. Amino acid sequences of homologous species were retrieved from the NCBI database (http://www.ncbi.nlm.nih.gov/datasets/gene/). The pathogenicity of the identified variants were predicted using MutationTaster (http://www.mutationtaster.org/), PolyPhen-2 (http://genetics.bwh.harvard.edu/pph2/), SIFT (http://https://www.sift-dna.org), CADD (https://cadd.gs.washington.edu), and REVEL (https://sites.google.com/site/revelgenomics/). The three-dimensional structure of PS (AF-P07225) from UniProt (http://www.uniprot.org) was used as a template. PyMOL (Version 3.0) was used to model, compare, and analyze the potential impact of *PROS1* variants on protein conformation.

## Results

### Phenotypic test results

The proband showed decreased PS: A (39%), TPS: Ag (53%), and FPS: Ag (42%). Similar reductions in PS: A, TPS: Ag, and FPS: Ag were observed in the proband’s mother, elder maternal aunt, and younger maternal aunt. The grandmother and elder male cousin also showed decreased PS: A and FPS: Ag. No significant abnormalities were detected in PLG: A, AT: A, and PC: A. Detailed results are provided in Table 1.

**Table 1:** Coagulation phenotype and genotype analysis of the probands and family members

Family members	Age (years)	PLG: A(%)	AT: A(%)	PC: A(%)	PS: A(%)	TPS: Ag (mg/L)	FPS: Ag (mg/L)	genetype c.1544G > A	genetype c.2001 A > G
Grandfather(Ⅰ_1_)	82	98	104	97	81	98	85	wild type	heterozygote
Grandmother(Ⅰ_2_)	80	101	103	89	56	65	45	heterozygote	wild type
Father(Ⅱ_1_)	59	90	110	106	88	96	83	wild type	heterozygote
Mother(Ⅱ_2_)	57	98	101	96	48	62	53	heterozygote	wild type
Uncle(Ⅱ_3_)	55	106	111	107	96	121	98	wild type	wild type
Elder maternal aunt(Ⅱ_4_)	53	105	99	107	54	57	41	heterozygote	wild type
Elder maternal uncle(Ⅱ_5_)	56	101	112	102	101	114	96	wild type	wild type
Younger maternal aunt(Ⅱ_6_)	50	101	103	112	42	51	38	heterozygote	heterozygote
Younger maternal uncle(Ⅱ_7_)	51	100	102	105	96	109	92	wild type	wild type
Sister(Ⅲ_1_)	34	101	112	125	74	97	81	wild type	heterozygote
Proband(Ⅲ_2_)	31	98	107	114	39	53	42	heterozygote	heterozygote
Brother(Ⅲ_3_)	27	87	102	112	94	106	91	wild type	wild type
Elder male cousin(Ⅲ_4_)	25	97	115	103	49	63	50	heterozygote	wild type
Younger male cousin(Ⅲ_5_)	24	103	110	107	66	89	82	wild type	heterozygote
Reference range		80∼120	98∼119	70∼130	65∼135	60∼140	80∼120		

Note: *PLG* A, plasminogen activity, *AT* A, antithrombin activity, *PC* A, protein C activity, *PS* A, Protein S activity, *TPS* Ag, total protein S antigen, *FPS* Ag, Free protein S antigen

### Thrombin generation and inhibition test

**Fig. 3:**
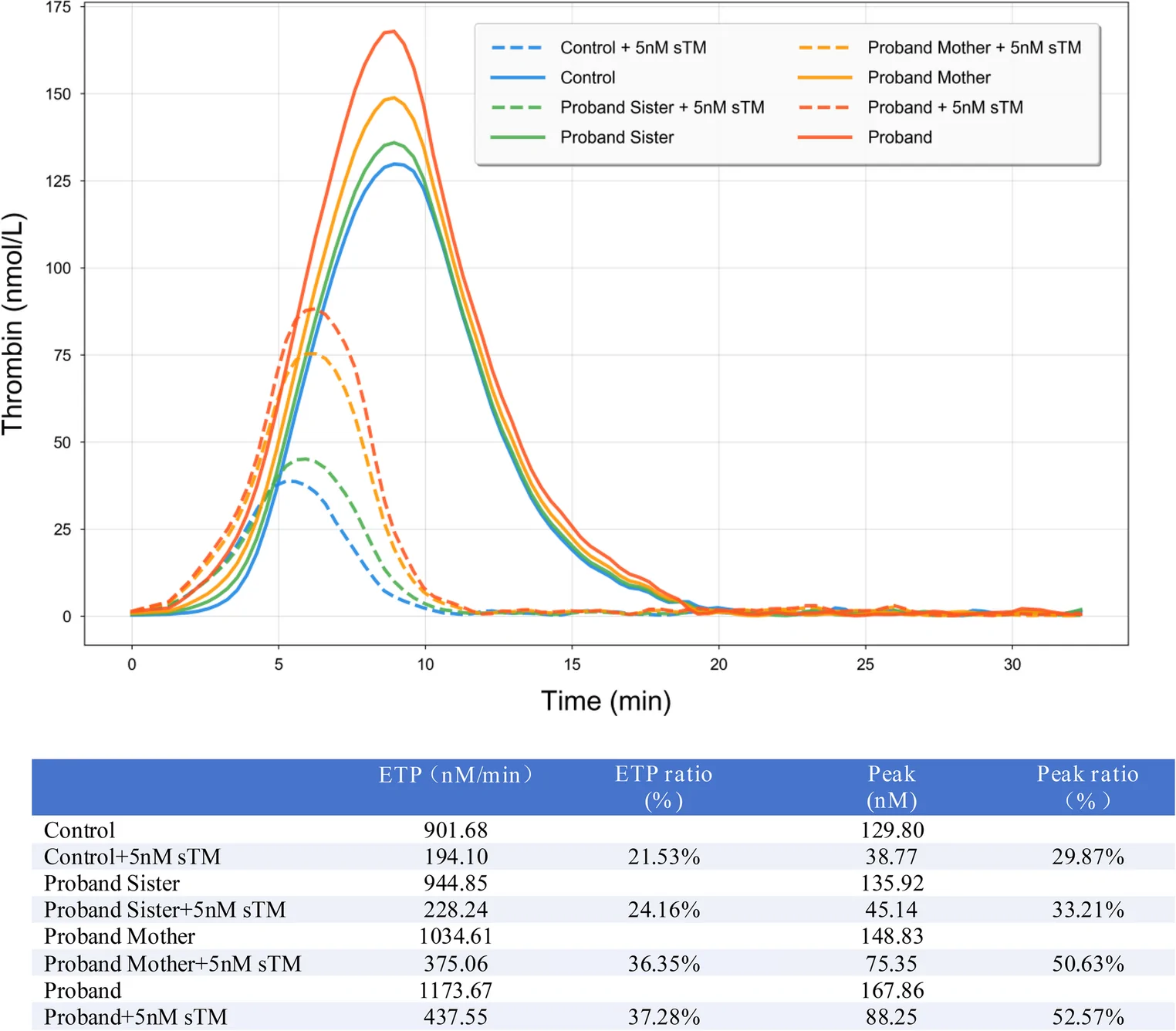
Thrombin generation and inhibition analysis in the proband with hereditary PSD and their family members. Note: sTM, soluble Thrombomodulin

Compared with normal plasma, the proband, proband sister, and proband mother demonstrated increased ETP and peak thrombin. The most notable elevation was displayed by the proband. In the presence of 5 nmol/L sTM, both parameters remained significantly elevated. The inhibitory effect of sTM on thrombin generation was reduced in the proband, proband sister, and proband mother (Fig. 3).

### Genetic testing results and pedigree analysis

**Fig. 4:**
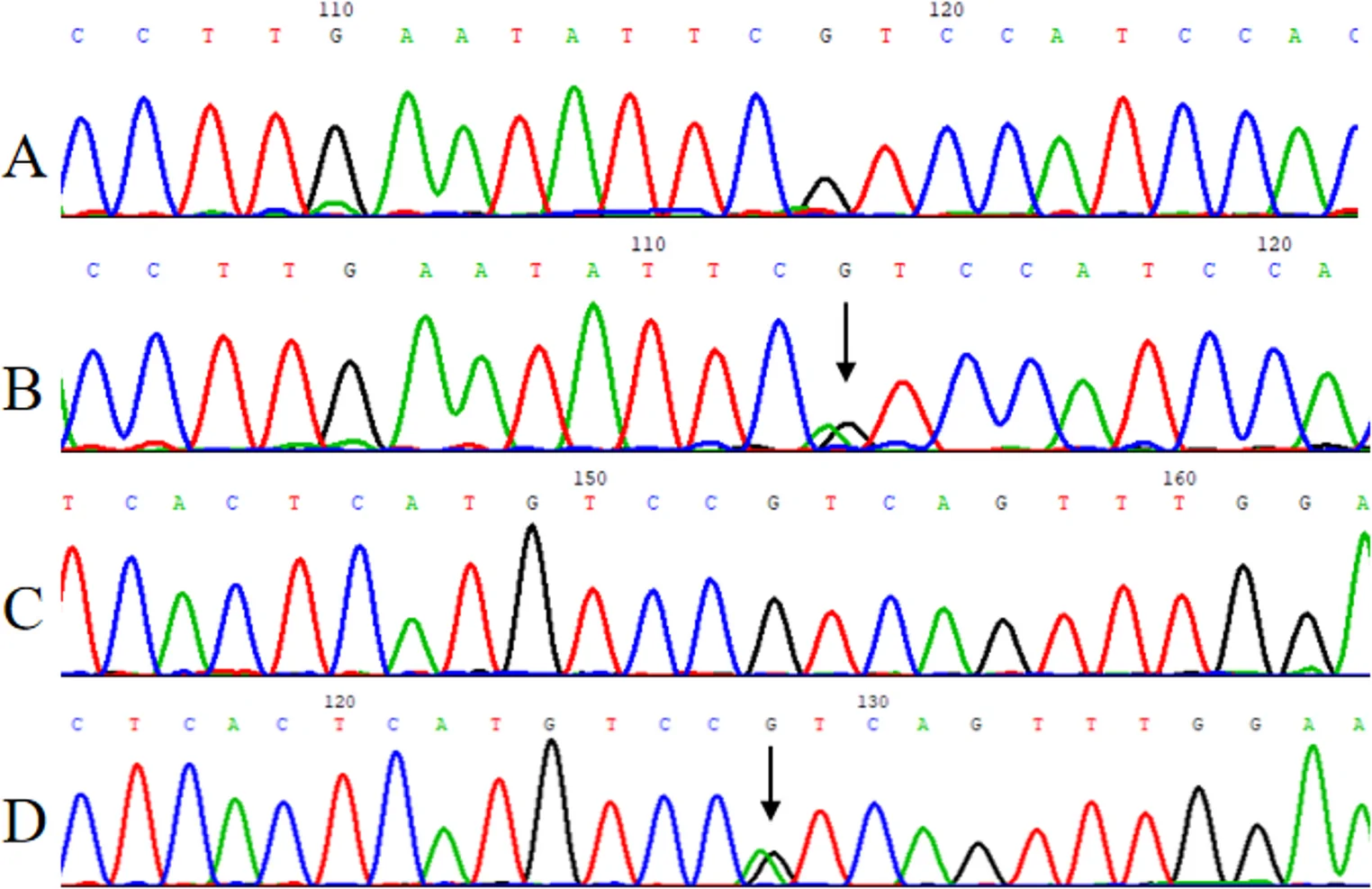
Sequencing results of the *PROS1* gene in the proband with hereditary PSD. **A**: Wild-type electropherogram showing base c.1544 in exon 13. **B**: Heterozygous missense variant c.1544G > A (arrow) in exon 13 of the proband. **C**: Wild-type electropherogram showing base c.2001 in exon 15. D: Heterozygous synonymous variant c.2001 A > G (arrow) in exon 15 of the proband.

The proband carried a heterozygous missense alteration c.1544G> (p.Arg515His) in exon 13 of *PROS1*, located in the LG2 functional domain of PS, and a heterozygous synonymous variant c.2001 A > G (p.Pro667Pro) in exon 15. The missense variant was present in the grandmother, mother, maternal elder aunt, and elder male cousin, while the synonymous variant was found in the grandfather, father, sister, and younger male cousin. The maternal younger aunt carried both variants, consistent with compound heterozygosity. All other family members showed wild-type genotypes (see Fig. 4). Database analysis using the Human Gene Mutation Database (HGMD; http://www.hgmd.cf.ac.uk/docs/login.html) and the 1000 Genomes Project (http://www.1000genomes.org/) showed that c.2001 A > G is a genetic polymorphism (allele frequency of 0.4188 [7]), whereas c.1544G > A has not been previously reported.

### Bioinformatics analysis results

**Fig. 5:**
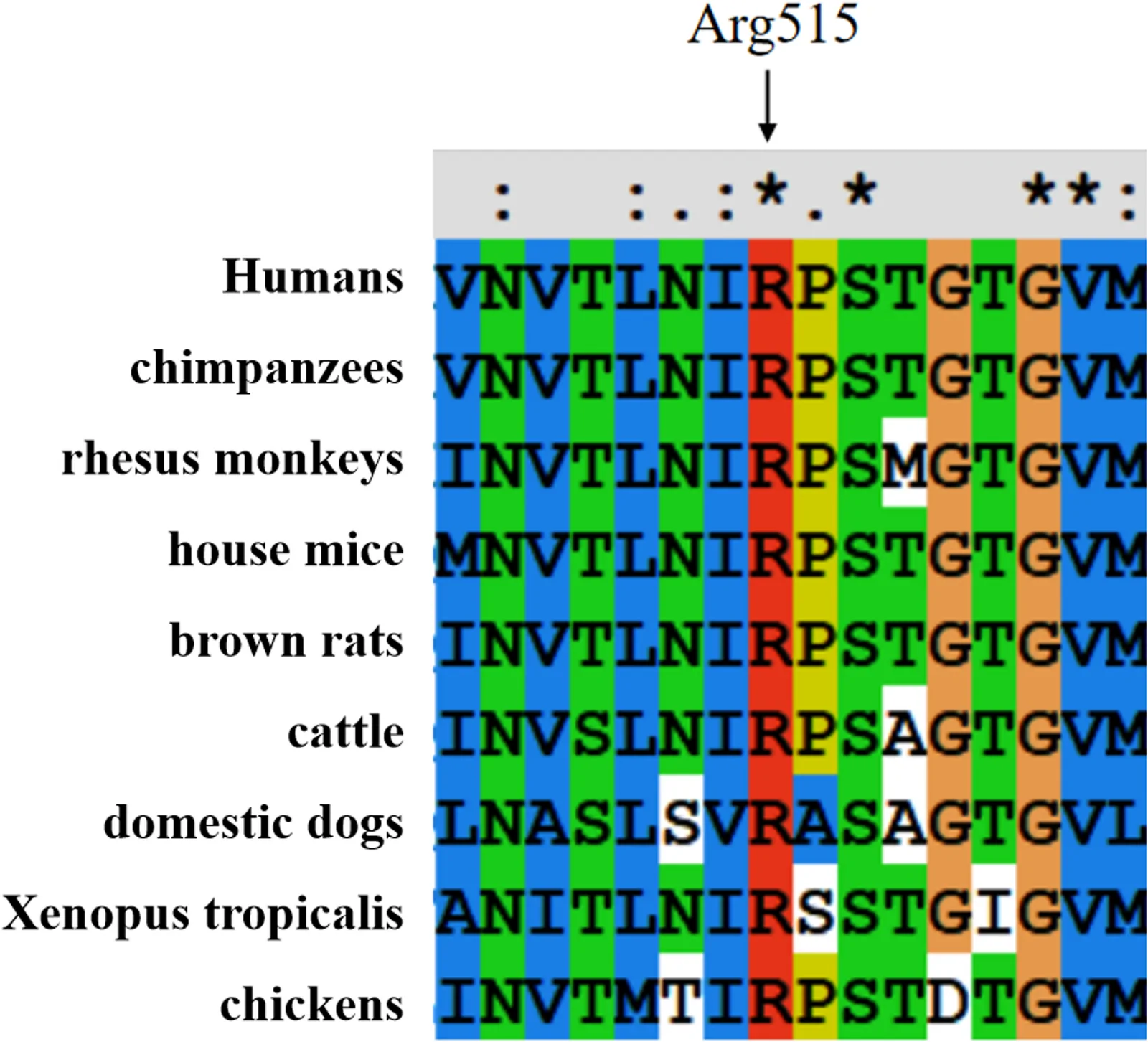
Multiple sequence alignment of homologous sequences at the Arg515 site. Note: * indicates fully conserved residues;: indicates highly conserved residues. indicates weakly conserved residues; blank indicates non-conserved residues

**Fig. 6:**
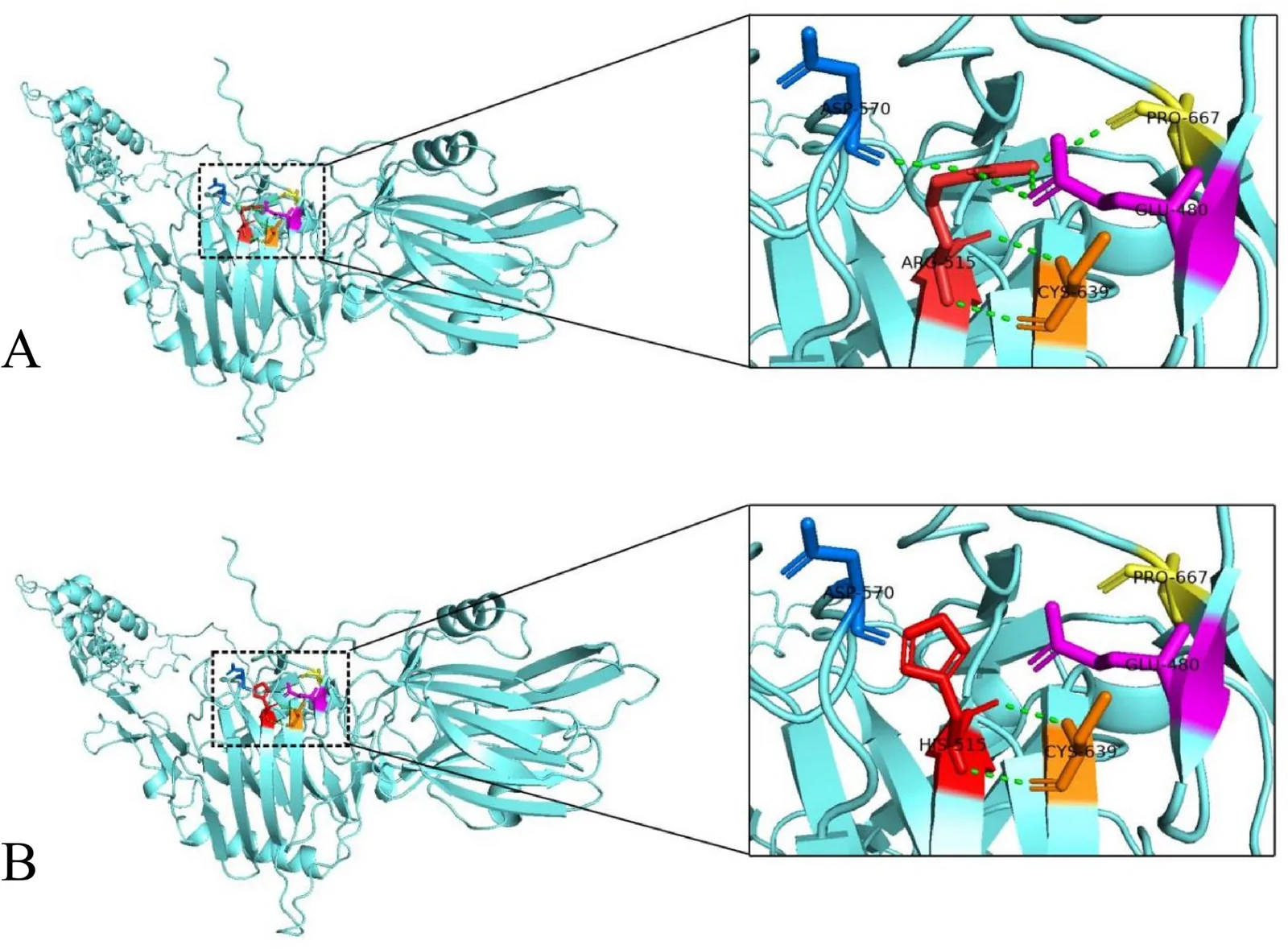
Structural modeling of PS. **A**: Wild-type PS. **B**: p.Arg515His mutant (hydrogen bonds shown in green)

ClustalX-2.1showed that the Arg515 residue is fully conserved among nine homologous species (Fig. 5). The p.Arg515His variant was predicted to be disease-causing (score 0.999) by mutationTaster, Non-pathogenic (score 0.068) by PolyPhen-2, disease-causing (score 0.04) by SIFT, disease-causing (score 23.3) by CADD, and Non-pathogenic (score 0.607) by REVEL. Protein structural modeling indicated that in wild-type PS, the polar residue Arg515 forms six hydrogen bonds with Glu480, Asp570, Cys639, and Pro667. Following the substitution of His515, the protein polarity remained unchanged. However, only two hydrogen bonds with Cys639 were retained, accompanied by the introduction of an imidazole ring. These changes may reduce local structural stability and alter protein conformation (Fig. 6).

## Discussion

Hereditary PSD is classified into three types. Type I is characterized by decreased PS: A, TPS: Ag, and FPS: Ag. Type II is defined by reduced PS: A with normal TPS: Ag and FPS: Ag, while Type III presents with reduced PS: A and FPS: Ag with normal TPS: Ag. Types I and III together account for approximately 95% of cases and are considered distinct phenotypic expressions resulting from pathogenic variants in the same gene. The proband in this study showed concurrent reductions in PS: A, TPS: Ag, and FPS: Ag, consistent with a diagnosis of type I hereditary PSD. Thrombin generation and inhibition assays further confirmed impaired anticoagulant activity in the proband’s plasma.

Studies have shown that homozygous or compound heterozygous variants in *PROS1* may cause severe conditions such as neonatal purpura fulminans, whereas heterozygous variants are associated with an increased risk of VTE and pulmonary embolism [8]. The proband carried the p.Arg515His variant together with the p.Pro667Pro polymorphism but did not develop extensive microthrombosis. This may be related to the p.Pro667Pro polymorphism, which is not associated with increased VTE risk but has been linked to reduced FPS: Ag levels [9]. In this pedigree, the maternal grandfather, father, elder sister, and younger male cousin carried this polymorphism, and their FPS: Ag levels were near the lower limit of the normal range, consistent with previous reports. These findings suggest a potential modulatory effect of this polymorphism on disease severity.

The p.Arg515His missense variant is located within the LG2 structural domain of the SHBG-like region of PS, a known hotspot for *PROS1* variants in the Han population [10]. As a non-enzymatic cofactor of TFPI, PS binds TFPI Kunitz 3 *via* the SHBG domain, enhancing TFPI-mediated inhibition of activated factor X (FXa) [11]. The Arg515 residue is highly conserved across species, and MutationTaster, SIFT and CADD predicted the variant to be pathogenic. Structural modeling showed that wild-type Arg515 forms six hydrogen bonds. Following substitution with histidine, only two hydrogen bonds with Cys639 were retained, and an imidazole ring was introduced. These changes may reduce electrostatic interactions and hydrogen bonding, leading to decreased protein stability. Previous studies have reported that amino acid substitutions at this site impair secretion of recombinant PS [12], and in vitro analysis of p.Arg515Cys has demonstrated intracellular degradation leading to secretion deficiency [13].

In this pedigree, the maternal grandmother, mother, eldest maternal aunt, and elder male cousin carried the p.Arg515His variant. The mother and eldest maternal aunt showed concurrent reductions in PS: A, TPS: Ag, and FPS: Ag, whereas the maternal grandmother and elder male cousin showed reduced PS: A and FPS: Ag with borderline TPS: Ag levels. These findings suggest that the p.Arg515His variant disrupts SHBG domain conformation and impairs PS secretion, representing a major pathogenic mechanism underlying type I hereditary PSD in this pedigree.

PSD is a hereditary, autosomal-dominant disorder with incomplete penetrance and variable expressivity. Type I inherited PSD is a well-established independent risk factor for venous thromboembolic events (VTE). CVST is a rare thrombotic disorder with a complex etiology, multiple predisposing factors, a predilection for young women, and frequent coexistence of acquired and inherited thrombophilic conditions. Common acquired risk factors include pregnancy, oral contraceptive use, malignancy, and autoimmune disorders. Thrombotic events did not occur in family members with the same gene mutation, suggesting that the proband’s CVST may be associated with long-term oral contraceptive use. The common manifestations of CVST include headache (74%-90%), focal neurological deficits (20%-50%), and epileptic seizures (20%-40%) [14]. MRV is considered the gold standard for CVST diagnosis, as it clearly demonstrates venous sinus filling defects and allows evaluation of venous outflow abnormalities [15].

The proband presented with abdominal pain, diarrhea, dizziness, headache, and right retro-orbital pain. Brain MRI revealed right temporal lobe lesions suggestive of venous infarction with hemorrhage. Following emergency management for intracranial hypertension and infection prophylaxis, the patient was transferred to the intensive care unit with a provisional diagnosis of intracranial hemorrhage and a suspected intracranial mass lesion. Subsequent MRV confirmed right-sided occlusive thrombus involving the superior internal jugular vein, sigmoid sinus, and transverse sinus, establishing a definitive diagnosis of CVST.

Guidelines from the American Heart Association and the European Stroke Organization recommend prompt anticoagulation following confirmed CVST to prevent thrombus propagation and facilitate recanalization. Acute treatment generally involves low-molecular-weight heparin, followed by long-term oral anticoagulation with vitamin K antagonists, while direct oral anticoagulants such as rivaroxaban and dabigatran are increasingly used as alternatives. The proband was managed in accordance with these recommendations. Due to intracranial hemorrhage in the acute phase, low-molecular-weight heparin (0.2 mL every 12 h) was administered under close monitoring, along with intravenous methylprednisolone for anti-inflammatory and anti-edema therapy, mannitol for intracranial pressure reduction, and levetiracetam for seizure prophylaxis. CT re-evaluation after 2 days showed partial resolution of the hemorrhage, and the heparin dose was increased to 0.4 mL every 12 h. After 16 days, imaging demonstrated partial thrombus resolution without hemorrhage progression, and anticoagulation was transitioned to oral rivaroxaban (10 mg twice daily). The patient was discharged on long-term anticoagulation and outpatient follow-up.

In conclusion, analysis of this hereditary PSD pedigree identified compound heterozygous *PROS1* variants p.Arg515His and p.Pro667Pro as contributors to reduced PS levels. The p.Arg515His variant is novel. Hereditary PSD likely contributed to CVST susceptibility, while disease manifestation is influenced by both genetic and environmental factors. Systematic thrombophilia screening and genetic testing are recommended in young patients with unexplained VTE to enable early diagnosis and familial prevention.

## Data Availability

All data generated or analysed during this study are included in this published article. The datasets used and/or analyzed in the current article are available from the corresponding author upon reasonable request.
